# Missed opportunities for HIV testing among newly diagnosed HIV-infected adults in Abidjan, Côte d'Ivoire

**DOI:** 10.1371/journal.pone.0185117

**Published:** 2017-10-04

**Authors:** Maxime Inghels, Serge Niangoran, Albert Minga, Jean Michel Yoboue, Lambert Dohoun, Abo Yao, Serge Eholié, Xavier Anglaret, Christine Danel

**Affiliations:** 1 Centre Population et Développement (CEPED), UMR 196 Paris Descartes–Institut de Recherche et Développement (IRD), France; 2 Programme PAC-CI/ANRS Research Site, CHU de Treichville, Abidjan, Côte d’Ivoire; 3 CMSDS, Centre Médical de Suivi des Donneurs de Sang, CNTS, Abidjan, Côte d’Ivoire; 4 Department of Infectious Diseases, Treichville Hospital, Abidjan, Côte d’Ivoire; 5 Centre Inserm 1219, Bordeaux University, Bordeaux, France; Hôpital Bichat-Claude Bernard, FRANCE

## Abstract

**Background:**

HIV testing is crucial for starting ART earlier in HIV-infected people. We describe Missed Opportunities (MO) for HIV testing among adults newly diagnosed with HIV in Abidjan, Côte d’Ivoire.

**Methods:**

Between april,2^nd^ 2013 and april 1st 2014, a cross-sectional study was conducted among all adults newly diagnosed (< 1year) for HIV at the Blood Donors Medical Center of Abidjan with face to face questionnaire. An MO for HIV testing was defined as a medical consultation for a clinical indicator (e.g. symptoms, hospitalization, and pregnancy) or a non-clinical indicator (e.g. high-risk sexual behavior, HIV-infected partner) potentially related to an HIV infection but did not lead to HIV test proposal by a health care professional.

**Results:**

Of the 341 patients who attended the center suring this period, 273 (157 women and 116 men) were included in this analysis. 130 (47.6%) reported at least one medical consultation for an indicator relevant for a test proposal between 1 month and five years prior to their diagnosis. Among them, 92 (77.3%) experienced at least one MO for testing. The 273 included patients reported a total of 216 indicators; 146 (67.6%) were reported without test proposal and thus were MO. Hospitalization, extreme lose of weight, chronic or repeat fever and herpes zoster were the indicators with the largest number of MO. While 66 (24.2%) patients experienced non-clinical indicators relevant to risk of HIV infection, only 11 (4.0%) mentioned it to a health professional.

**Conclusion:**

MO for HIV testing are frequent, even in situations for which testing is clearly recommended. Better train healthcare professionals and creating new opportunities of testing inside and, outside of medical settings are crucial to improve HIV control.

## Introduction

Early antiretroviral treatment initiation has both individual benefits, through reduction of the risk of HIV-related morbidity and mortality [1,2], and collective benefits, through reduction of the risk of HIV transmission [3,4]. Recent World Health Organization guidelines recommend initiating unconditional treatment (i.e. regardless of CD4 count) for all people living with HIV (PLHIV) [5]. However, many PLHIV are diagnosed at an advanced stage of infection. In sub-Saharan Africa, an estimated 45–70% of PLHIV are screened with less than 200 CD4/mm3 [6–8]. Early treatment cannot be achieved without early screening.

Given the need to reduce the time between HIV infection and diagnosis, several studies have examined the healthcare pathway prior to diagnosis to identify opportunities for health professionals to offer HIV tests. These studies, mainly conducted in northern countries, show that almost all PLHIV receive some type of health care in the years before diagnosis, and many of them have clinical indicators (e.g., sexually transmitted infections, chronic diarrhea, tuberculosis) and/or non-clinical indicators (e.g., high-risk behaviors or membership in a high-risk group) that are potentially associated with HIV [9–15]. However, the number of HIV tests offered by health professionals based on these indicators remains insufficient [9–15]. To the best of our knowledge, only two studies have documented the healthcare pathway prior HIV diagnosis among newly diagnosed PLHIV in the African context, and neither fully describes the history of HIV testing opportunities among participants [8,16].

In Côte d’Ivoire, one of the West African countries hit hardest by the HIV epidemic [17], no study has documented the care pathway prior to diagnosis, and most PLHIV are diagnosed at advanced disease stage with low CD4 counts (<200 CD4/mm^3^) [7,18]. In light of this, we conducted a study of newly diagnosed PLHIV in order to characterize missed opportunities for testing and identify types of opportunities that are often missed.

## Materials and methods

### Study design and site

We conducted a cross-sectional study among newly diagnosed PLHIV at the Blood Donors Medical Center (*Centre Médical de Suivi des Donneurs de Sang*-CMSDS) located in Abidjan, Côte d’Ivoire. CMSDS is part of the National Blood Transfusion Center of Abidjan, which coordinates all blood donations in Côte d’Ivoire. The CMSDS receives people for voluntary HIV testing, and also serves as a comprehensive HIV care center to which PLHIV can be referred after diagnosis.

This study was supported by the French Agency for Research on AIDS and Viral Hepatitis B and C (ANRS) and integrated into a larger project: ANRS 12 277 PRECO-CI. A component of this latter project is focused on “missed opportunities for HIV screening,” with the aim of describing missed opportunities for HIV testing in patient history among newly diagnosed individuals when they first visit the CMSDS.

### Study population

For one year, all adults (age>18 years) visiting the CMSDS for the first time were asked to participate in this study. Only patients who agreed to participate and who had been diagnosed with HIV within the year prior to their visit were included in the study. The study population included blood donors diagnosed with HIV at the CMSDS, patients diagnosed at the voluntary testing center of the CMSDS and patients diagnosed at other health facilities and referred to the CMSDS during the study period.

### Data collection

Patients underwent a physical examination upon inclusion in the study. Routine laboratory tests were administered, including a hemoglobin test and CD4 cell count. The Ivoirian government provides laboratory analysis and antiretroviral therapy at no cost to patients.

Clinical and non clinical indicators, sociodemographic and clinical data were collected through standardized face-to-face questionnaires administered by health professionals. We recorded two main categories of data: clinical indicators and non-clinical indicators that occurred during the 5 years prior to diagnosis. For both clinical and non-clinical indicators, we recorded whether the patient proceeded with a test or refused a test. For non-clinical indicators such as been a membership in a high-risk group or knowing his sexual partner as HIV-positive, we recorded whether the patient having mentioned this indicator once to a professional health care and whether at least one test was proposed for this indicator. We also recorded circumstances of the HIV-positive status discovery and information on previous HIV tests.

### Definition of the variable of interest

We defined a testing opportunity as a clinical or non-clinical indicator occurring in the period between 1 month and 5 years prior to the first HIV diagnosis which should have prompted an HIV test. Clinical indicators included: (i) consultation for an opportunistic infection or symptom potentially related to HIV, (ii) hospital admission for at least one night and (iii) pregnancy, with at least one prenatal visit and/or giving birth in a health center. Non-clinical indicators included (i) high-risk sexual behaviors (e.g., unsafe sex with a partner of unknown HIV status), (ii) membership in a high-risk group (e.g., injection drug users, men who have sex with men), and (iii) potential indicators of an infection among people in close contact with the patient (e.g., diagnosed HIV infection, frequent illness, or death suspected to be attributable to HIV infection among a patient’s partners or children).

A missed opportunity for testing was defined as a testing opportunity where the patient did not reported a test proposal by a professional healthcare.

During the study period, the majority of health facilities have access to rapid HIV screening tests except few private health structures with results rendering in several days. Thus, we assume that one-month delay was sufficient in the Ivorian context for proceed an HIV test and obtain the result after HIV related indicator.

### Statistical analyses

We present qualitative variables as percentages, and quantitative variables with medians and interquartile ranges (IQR). We use the following statistical tests: Chi-square test or Fisher’s exact test to compare qualitative variables and the Wilcoxon rank-sum test to compare the medians of a quantitative variable between groups. Because of overdispersion we use a negative binomial regression to describe CD4 count at diagnosis by number of missed opportunities experienced. All analyses were conducted using *R* software (Version 3.3.2).

### Ethical considerations

This study was approved by the National Ethics Committee for Health Research of Côte d'Ivoire. Health professionals gave information about the study to each patient prior to their inclusion. After reading the information notice, each patient who wished to enroll in the study signed an informed consent form. All data collected were encoded to ensure the anonymity of patients recruited for the study.

## Results

### Study population

Between April 2, 2013 and April 1, 2014, 341 patients visited CMSDS for their first time. Of these, 301 people (88%) agreed to participate in this study. The 40 patients who refused to participate were not significantly different from patients who agreed to participate in terms of age, sex, baseline CD4 count, and the reason for the test that led to HIV diagnosis (Table 1). Of the 301 people who agreed to participate, 28 were excluded because their HIV diagnosis occurred more than a year prior to data collection. Excluded individuals were not significantly different from non-excluded patients in terms of age and sex but have a lower baseline median CD4 count at HIV diagnosis (165/mm^3^ vs. 265/mm^3^, p = 0.01). Among included patients, the median time [IQR] between HIV diagnosis and questionnaire completion was 19 [11–27] days.

**Table 1:** Individual characteristics among enrolled patients (n = 301) and those who refused to participate in the survey (n = 40).

		Enrolled (N = 301)		Refused to participate (N = 40)		*p-value*
		N	%	N	%	
**Sex,** female		178	59.1	22	55.0	*0*.*74*
**Age** years, median [IQR]		38	[32–45]	38	[34–44]	*0*.*59*
**CD4** /mm^3^, median [IQR]		254	[110–437]	296	[116–407]	*0*.*80*
**WHO stage**,						
	1	146	57.9	20	60.6	*0*.*36*
	2	60	23.8	11	33.3	
	3	35	13.9	2	6.1	
	4	11	4.4	0	0.0	
**Registred as a Blood donor**, yes		73	24.3	8	20.0	*0*.*82*

IQR: interquartile range

n.b.: Percentages presented in the table and taken into account for missing values which were found for WHO stage (49 missing values among enrolled patients and 7 among those who refused to participate), CD4 count (10 missing values among enrolled patients and 3 among those who refused to participate).

P value test (Chi-square test or Fisher’s exact test to compare qualitative variables and the Wilcoxon rank-sum test to compare the medians of a quantitative variable between groups)

The clinical, biological and sociodemographic characteristics of the 273 patients (157 women and 116 men) included in this analysis are detailed in Table 2. On average, male participants had a higher level of education and were significantly older, more often financially independent, more likely to be married or living with a partner, and more likely to report multiple sexual partners than women. There was not a significative difference in median CD4 count between men and women (254/mm^3^ vs. 270/mm^3^, p = 0.19).

**Table 2:** Characteristics (clinical, social, HIV testing history, clinical and non-clinical indicators) of patients recently HIV diagnosed at the CMSDS, according to sex (N = 273).

			Women (N = 157)		Men (N = 116)		*p-value*
			N	%	N	%	
***Clinical and biological characteristics***							
	**Clinical stage,** WHO Stage 3,4		22	14.0	18	15.5	*0*.*85*
	**Body Mass Index** median, [IQR]		22.6	[19.5–26.6]	22.0	[19.0–24.2]	*0*.*05*
	**CD4 Count** /mm^3^, median, [IQR]		270	[137–477]	254	[106–436]	*0*.*19*
	**Hemoglobin** dl/mm^3^, median, [IQR]		11	[10–12]	12	[11–14]	*<0*.*01*
***Individual characteristics***							
	**Age** years, median, [IQR]		35	[30–42]	40	[34–48]	*<0*.*01*
	**Marital status**						*<0*.*01*
		Single	79	50.3	36	31.0	
		Married or living with a partner	78	49.7	80	69.0	
	**Education level**						*<0*.*01*
		No education or primary	78	49.7	28	24.1	
		Secondary or higher	79	50.3	88	75.9	
	**Financially independent**, yes		65	41.4	92	79.3	*<0*.*01*
	**Occupation**						*<0*.*01*
		Formal	41	26.1	69	59.5	
		Informal	98	62.4	36	31.0	
		Unemployed or others	18	11.5	11	9.5	
	**Number of sexual partners last month**						*<0*.*01*
		None	74	47.4	52	44.8	
		1	78	50.0	47	40.5	
		2 or more	4	2.6	17	14.7	
	**Has been pregnant during the past 5 years**, yes		13	19.7	-	-	
***HIV test and history of testing***							
	**Reason for the test that lead to HIV diagnosis**						*<0*.*01*
		Blood donation	27	17.3	42	36.5	
		Sickness	62	39.7	51	44.3	
		Testing without sickness or pregnancy^µ^	67	42.9	22	19.1	
	**Has already been tested at least once for HIV**, yes		57	36.3	32	27.6	*0*.*16*
	**Has already refused a test**, yes		11	7.0	13	11.2	*0*.*32*
	**Has already renounced to do the test**, yes		16	10.2	12	10.3	*1*.*00*
***Indicator in the last 5 years***							
	**History of hospitalization**		33	21.0	15	12.9	*0*.*15*
	**History of consultation for clinical symptom potentially related to an HIV infection**		46	29.3	40	34.5	*0*.*44*
	**History of pregnancy**						
		At least one with medical contact	30	19.1	-	-	
		None with medical contact	1	0.6	-	-	
	**History of non-clinical indicator**						
		At least one mentioned to an health professional	7	4.5	4	3.4	*0*.*37*
		None mentioned once to an health professional	35	22.3	18	15.5	*0*.*02*

^µ^ 3 women (1,9%) were diagnosed by a test performed during pregnancy

IQR: interquartile range

n.b.: Percentages presented in the table exclude missing values which were found for WHO stage (18 missing values among men and 24 among women), CD4 count (3 among men and 4 among women), Hemoglobin (3 among both men and women), raison of testing that lead to HIV diagnosis (1 among men and 1 among women), number of sexual partner (1 among women), having mentioned a non-clinical indicator to an health professional (2 among women).

### HIV testing and diagnosis characteristics

More than two thirds of participants had never been tested for HIV prior to the test that led to their positive diagnosis (Table 2). 24 patients (8.8%) had at least once refused a test and 28 (10.3%) had once expressed the willingness to perform a test but finally changed their mind during the five-year period preceding their diagnosis. Reasons for testing that led to HIV diagnosis included sickness (41.7%), voluntary testing without sickness (31.7%), blood donation (25.5%) and pregnancy (1.1%). Men were more likely to have discovered their HIV-positive status through blood donation (36.5% for men vs. 17.3% for women), but they were most likely to discover it through sickness (44.3% for men vs 39.7% for women) while women discovered their HIV-positive status most frequently through voluntary testing without sickness (19.1% for men vs 41.0% for women) (Table 2).

### Testing opportunities

**Fig 1:**
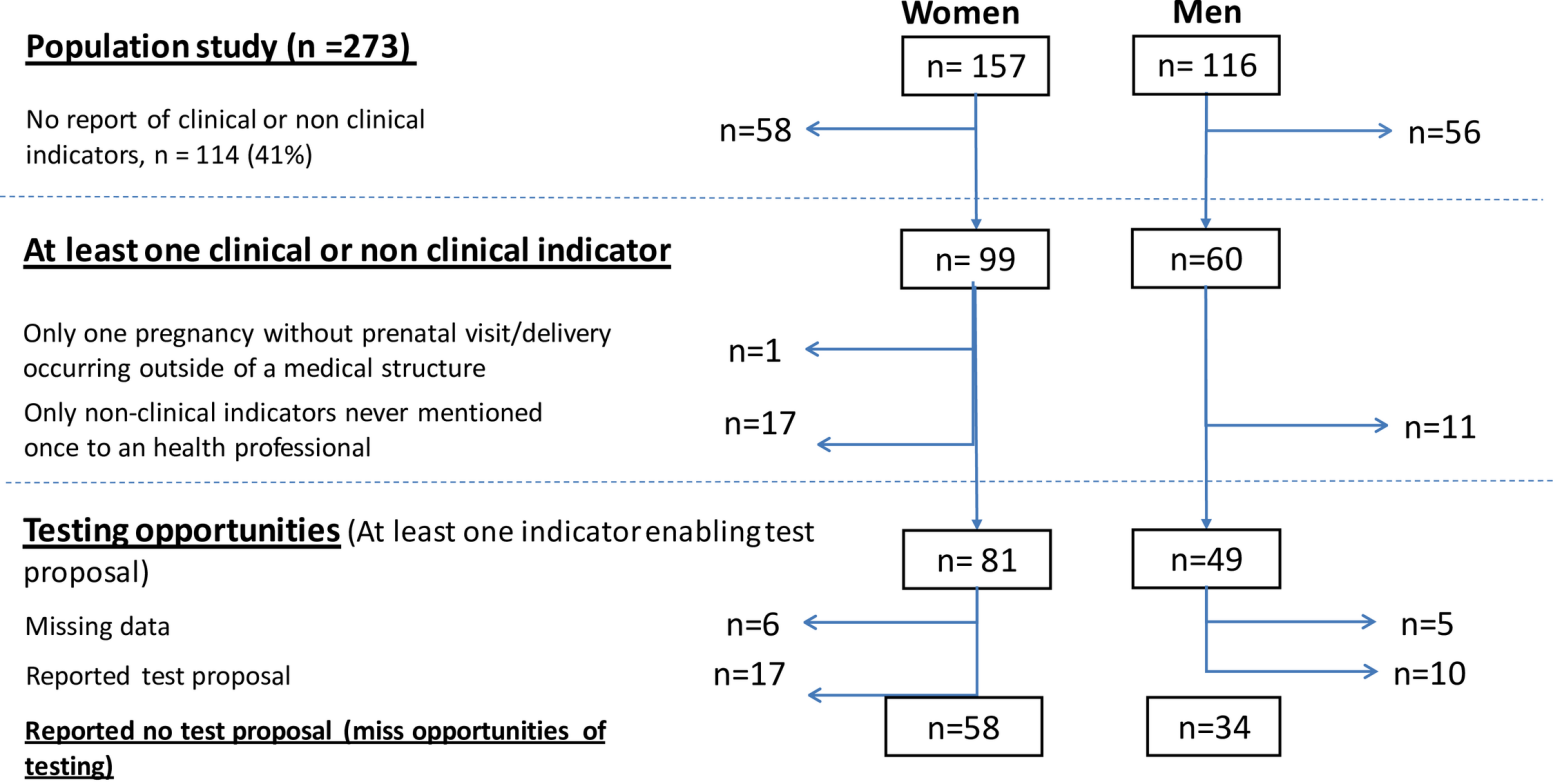
Flow chart of individuals newly diagnosed at the CMSDS, who experienced at least one missed opportunities for testing proposal during the last five years prior HIV-diagnostic, according to sex (n = 273).

Among our study population, 159 patients reported at least one clinical or non-clinical indicator during the 5 years prior to their diagnosis (Fig 1). Among this group, one woman reported only a pregnancy with delivery outside of medical structure and 28 persons reported only non-clinical indicators that had never been mentioned to a health professional. The remaining 130 patients (47.6%) reported at least one testing opportunity. Of the 130 patients who reported at least one opportunity of testing, 119 have data on test proposals for any of their clinical or non-clinical indicators. 77.3% reported at least one missed opportunity for testing during the 5 years prior their diagnosis, and 22.7% were thus tested negative or refused the test proposal.

### Description of the missed opportunities for testing

Overall, 159 patients reported 312 clinical and/or non-clinical indicators. The median time difference between these indicators and the HIV diagnosis was 17 [5–39] months. On the 312 indicators, 70 did not lead to a medical consultation and 26 did not have data on associated test proposals. Of the remaining 216 indicators that were testing opportunities (72 among men and 144 among women), 146 (67.6%) were reported without test proposal and thus were missed opportunities. There was no statistical difference between proportion of missed opportunities occurred among men and women (70.8% and 66.0% respectively, p = 0.57).

**Fig 2:**
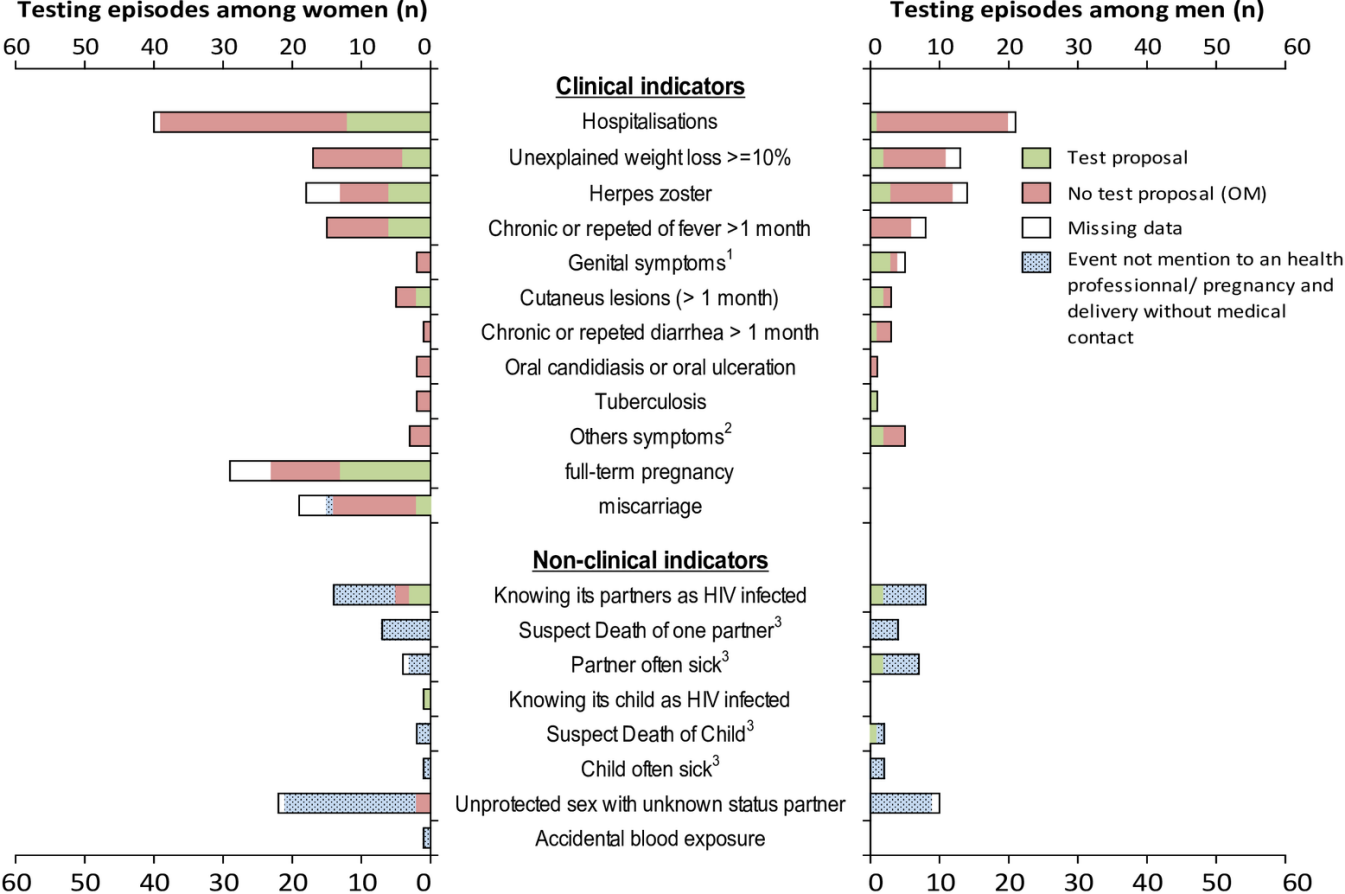
Description of the 308 clinical and non-clinical indicators occurred during the past 5 years prior diagnosis according to sex, in patients recently HIV diagnosed at the CMSDS (N = 159). ^1^ Genital symptoms ulcérations or Urethral discharge ^2^ others symptoms contained sinusitis (3 events, 100% MO), fungal nail infections (2 events, 100% MO), kidney failure (1 event, 0% MO), facial peripheric paralysis (1 event, 0% MO), Bacterial Pneumonia (1 event, 0% MO) ^3^ Not know as HIV infected.

The repartition of the different type of testing opportunities is presented in the Fig 2. 203 (94.0%) of testing opportunities were for a clinical indicator; 142 (70.0%) of these opportunities were missed. Hospitalization, unexplained weight loss, chronic or repeat fever and herpes zoster were the main clinical indicators of missed opportunities for testing.

Hospitalization episodes concentrate the higher number of missed opportunities for testing. Missed opportunities during hospitalization episodes occurred significantly more frequently among men than women (19 (95.0%) vs. 25 (69.4%), p = 0.04). However, this correlation was still positive but not significant when excluding the 8 hospitalizations for related pregnancy issues–caesarean section or ectopic pregnancy for example (19 (95.0%) vs. 21 (75.0%), p = 0.16). Reasons for hospitalization were mainly for medical symptoms (39.7% and 22.4% respectively for potential infectious and non-infectious disease), gynecological causes (20.7%) and other chirurgical matters (17.2%). Testing proposal was reported for 19.0%, 15.4%, 50.0%, and 0.0% patients respectively in these situations.

18 (54.5%) pregnancies were reported without at least one test proposal during prenatal visit and/or deliveries and were missed opportunities. Pregnancies that ended in miscarriage were more often but not significantly associated with missed opportunities for testing compared to full term pregnancies (8 (80.0%) vs. 10 (43.5%), p = 0.07).

Regarding non-clinical indicators, of the 82 non-clinical indicators reported, only 13 (15.9%) were mentioned to a health care professional and were thus actual testing opportunities. The two most common non-clinical indicators reported were (i) having unprotected sex and (ii) having a sexual partner known to be infected with HIV. None of our population reported being drug users or men who have sex with men.

### Missed opportunities for testing were associated with lower CD4 level at diagnosis

In Table 3, we present the clinical, biological and socio-demographic characteristics of patients who neither experienced clinical nor non-clinical indicators compared with those who experienced at least one clinical or non-clinical indicator, with or without MO for testing, for the 262 patients who have data on test proposals. Patients who experienced a missed opportunity for testing were more likely, to have lower median CD4 count, to be diagnosed at a later WHO disease stage, to have a lower median hemoglobin concentration and to have been diagnosed for HIV because of sickness.

**Fig 3:**
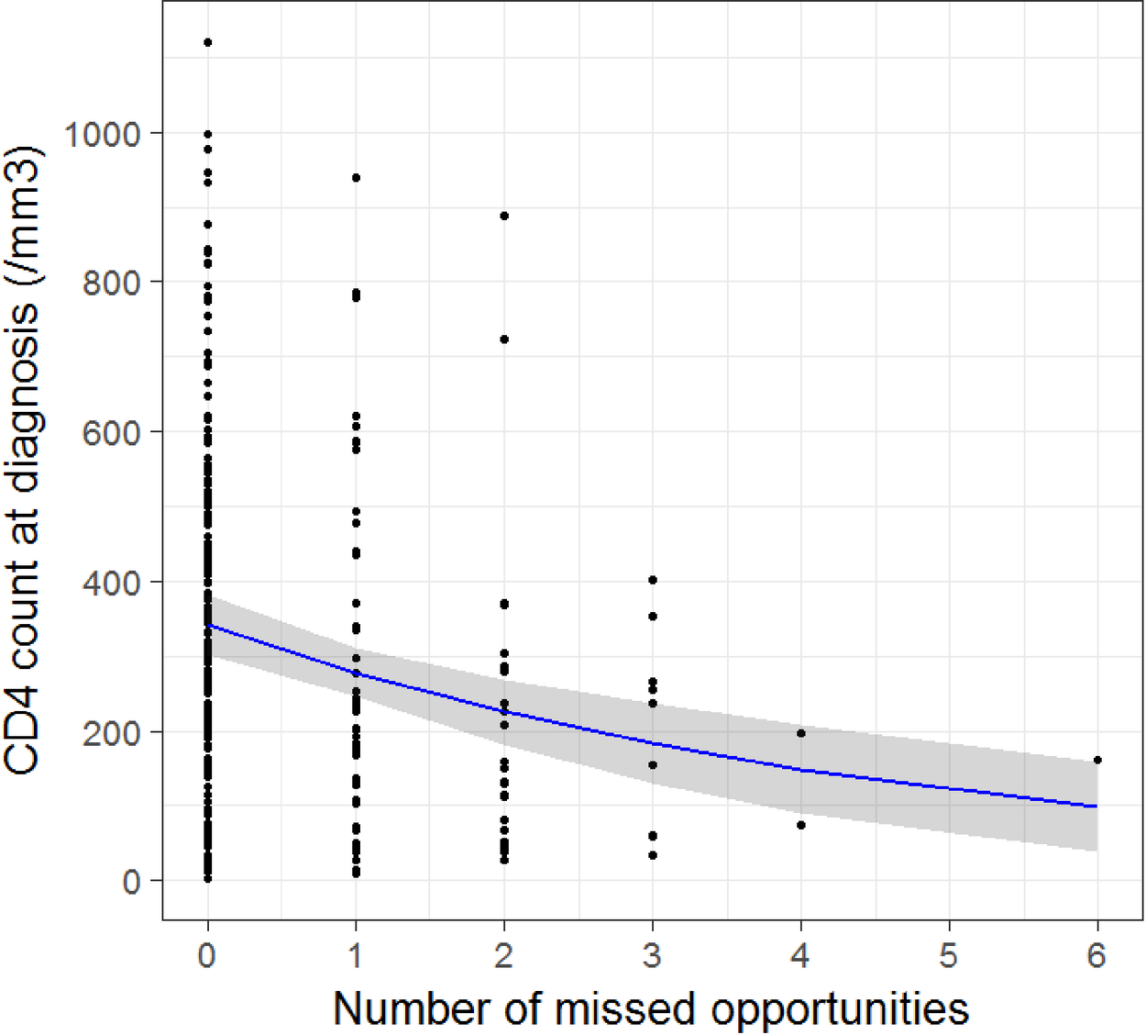
CD4 cell count at HIV diagnosis according to the number of missed opportunities for HIV test proposal occurred in the 5 years prior diagnosis among patients recently HIV diagnosed at the CMSDS, (negative binomial regression model)(N = 255*). *n.b.: among the 273 patients included in the study, 11 and 7 individuals were excluded because of missing value on test proposal and on CD4 count respectively.

CD4 cell count at diagnosis decreased significantly with increasing missed opportunities for testing (Fig 3). An augmentation of one missed opportunity was associated with a decrease of 19.6% of the number CD4/mm^3^ at diagnosis (IC_95%_[10.2%– 27.4%], p<0.001).

**Table 3:** Clinical, biological and socio demographic characteristics of patients recently diagnosed HIV positive who experienced no clinical nor non-clinical indicators compared with those who experienced at least one clinical or non-clinical indicator, with or without MO for testing (N = 262*).

			Clinical or non-clinical indicator not reported		Reported clinical or non-clinical indicator				*p-value* (global)
					With missed opportunities for testing		Without missed opportunities for testing		
			(N = 143)		(N = 92)		(N = 27)		
			N	%	N	%	N	%	
***Clinical and biological characteristics***									
	**Clinical stage,** WHO Stage 3,4		13	10.6	24	32.0	2	8.0	*<0*.*01*
	**Body Mass Index,** median, [IQR]		22.5	[20.3–24.9]	22.10	[18.7–25.4]	21.7	[19.8–25.4]	*0*.*51*
	**CD4 Count** /mm^3^, median, [IQR]		312	[180–507]	197	[73–340]	354	[96–473]	*<0*.*01*
	**Hemoglobin** (dl/mm^3^), median [IQR]		12	[11–13]	11	[10–12]	12	[11–13]	*<0*.*01*
***Individual characteristics***									
	**Sex**, female		76	53.1	58	63.0	17	63.0	*0*.*27*
	**Age** years, median, [IQR]		38	[32–46]	38	[31–45]	34	[30–39]	*0*.*23*
***HIV test and history of testing***									
	**Reason for the test that lead to HIV diagnosis**								*<0*.*01*
		Blood donation	59	41.5	6	6.6	3	11.1	
		Sickness	42	29.6	55	60.4	12	44.4	
		testing without sickness or pregnancy ^µ^	41	28.9	30	33.0	12	44.4	
	**Has already been tested at least once for HIV,** yes		46	32.2	25	27.2	14	51.9	*0*.*06*

^µ^ 3 women (1,9%) were diagnosed by a test performed during pregnancy

IQR: interquartile range

*n.b.: among the 273 patients included in the study, 11 individuals were excluded because of missing value on test proposal.

Percentages presented in the table exclude missing values which were found for WHO stage (20 missing values among individuals with no indicator, 17 missing values among individuals with MO and 2 among individuals without MO), CD4 count (4 missing values among individuals with no indicator, 1 missing values among individuals with MO and 1 among individuals without MO), Hemoglobin (4 missing values among individuals with no indicator, 1 missing values among individuals with MO and 1 among individuals without MO), number of sexual partner (1 missing values among individuals with no indicator) and raison of testing that lead to HIV diagnosis (1 missing values among individuals with no indicator and 1 missing values among individuals with MO).

p-value was performed among the three groups.

## Discussion

To the best of our knowledge, our study is one of the few studies to accurately detail the care pathway of a population of newly-diagnosed PLHIV in Africa. The two main findings of our study were the high proportion of missed opportunities for testing and the high proportion of PLHIV who experienced neither clinical nor non-clinical indicator in the five years prior to their diagnosis that could have led to a test proposal by a health care professional.

Hospitalization was one of the indicators with the highest rate of missed opportunity. Other studies have shown a high incidence of missed opportunities for testing within hospitals, particularly within unspecialized care units and emergency departments [13–15,19,20]. Although our study does not detail the types of hospital services associated with missed opportunities, these results suggest that establishment of routine HIV testing upon admission to a hospital should be considered as a mechanism for detecting HIV earlier. This measure has proven to be cost-effective even in contexts with lower HIV prevalence in the general population than is found in Côte d’Ivoire [21].

More than half of pregnancies were found to be missed opportunities for test proposal. This result is consistent with results of the last demographic health survey in Cote d’Ivoire, in which only 58% of women reported being offered an HIV test by a health care professional during their last pregnancy despite the recommendation of the Ivorian Ministry of Health for routinely offering HIV testing as part of prenatal care [22]. Missed opportunities for testing were more prevalent, not significantly because of lack of power, among pregnancies that ended prematurely, but it can be explained by the fact that they do not have the opportunity to have a test proposal compared to those who have full–term pregnancy. Regardless, the majority of pregnant women in Côte d’Ivoire receive prenatal care with a trained healthcare provider and should be offered HIV testing at that time [22]. More studies are needed to identify the reasons that HIV testing is not always offered during delivery and pre-natal consultation in Côte d’Ivoire.

Extreme weight loss, repeat fever and herpes zoster, symptoms commonly associated with HIV infection, represented a high number of missed opportunities for testing in this study. Other studies in northern countries have shown that HIV tests were offered less frequently in the indicator of skin diseases or significant weight loss compared to other diseases, such as sexually transmitted infections [9,11,15]. However, almost all studies on this topic have been conducted in Northern countries with comparatively lower HIV prevalence. In Côte d’Ivoire, where HIV prevalence in the general population is around 3.7%, symptoms like weight loss, repeat fever or skin diseases should automatically lead to proposal of an HIV test [23]. Barriers facing healthcare provider in testing proposal in case of these clinical indicator need to be assessed.

In our sample, men and women experienced the same proportion of missed opportunities for test proposal. These results differ from other studies that have found women to experience missed opportunities for testing more frequently than men, although the context of these other studies (Northern countries with epidemics concentrated in MSM) is not comparable to the Ivoirian context [15,20]. Nevertheless, we did identify some differences between men and women: men are less likely than women to be tested for HIV during hospitalizations, and women have frequent missed opportunities for testing during pregnancy.

The most significant novel contribution of this study is the documentation of previous non-clinical indicators that may have been indicators of HIV infection among people newly diagnosed with HIV. These non-clinical indicators include circumstances such as having a partner with HIV or the death of a partner or child that is suspected to be due to HIV infection. Less than a quarter of our study population reported a non-clinical indicator that should have prompted a test proposal. However, non-clinical indicators were largely under-reported to a healthcare professional and it is also possible that non-clinical indicators may often escape the attention of healthcare providers during consultations. Assessment of sexual risk behaviors among patients often appears to be inadequate among healthcare providers in other contexts [9,10,12]. Reluctance on the part of patients or health care professionals to discuss sensitive non-clinical indicators may present an ongoing challenge to effective risk evaluation.

42% of our study population reported no clinical or non-clinical indicators in the five years prior to diagnosis, highlighting the need for routine testing before patients present to care with symptoms suggestive of HIV. Currently, healthcare providers are not able to reach all PLHIV at an early stage. A study conducted in Uganda among 612 PLHIV shows that only 21% reported having already seen a physician and 11% reported been hospitalized in the period prior to their diagnosis [8]. In our study, which included a predominantly urban population, people likely have better access to medical care compared to the general population in Côte d’Ivoire. Sustained efforts to increase testing outside of health facilities (e.g., HIV testing campaigns, community-based HIV testing) are crucial to increasing testing opportunities. This may be especially important for men, who reported fewer indicators for testing opportunities than women, who had more frequent encounters with the health system as part of pregnancy or health care for their children.

Our results show that PLHIV with several missed opportunities were diagnosed at an immunocompromised stage with more severe anemia (i.e. lower hemoglobin level), compromising their life span [24]. Patients in our study were diagnosed very late with low CD4 level and majority was diagnosed because of sickness. It is a real loss of chance for the effectiveness of the treatment and highlights the need for earlier testing intervention.

Our study has several limitations, including recall bias. The 5 year-period prior to HIV diagnosis for the occurrence of clinical indicators may have caused us to underestimate the number of clinical and non-clinical indicators, opportunities for testing, test proposals and missed opportunities of testing.

The questionnaires were face-to-face administered and this may lead to a desirability bias, in particular for intimate topics (non-clinical indicators), and then undersestimate the proportion of MSM or IDU in our population study. In addition, our sample is likely not representative of Côte d’Ivoire’s entire PLHIV population. One third of our sample were blood donors, who may have better uptake of testing in the period before their diagnosis and thus may be diagnosed at a relatively early stage of infection. In fact in other studies, the average CD4 at diagnosis among PLHIV is documented at a lower stage in Côte d’Ivoire and in West Africa, suggesting that late diagnosis and missed opportunities are likely to be more frequent on average than in our study population [7].

Missed opportunities for testing were very common among our sample and may be even more frequent among the full population of PLHIV in Côte d’Ivoire. In a setting where the HIV epidemic remains a major public health problem, early ART and early testing of PLHIV cannot be achieved without: (i) offering testing with greater frequency in clinical situations (e.g., hospitalizations, pregnancy), (ii) testing in response to HIV-related diseases or symptoms, (iii) conducting in-depth investigation of patients’ non-clinical indicators (e.g., HIV-infected partner, deceased partner) with offers of follow-up testing when appropriate, and (iv) creating new opportunities of testing inside and outside of health facilities to reach the sizeable proportion of PLHIV with no indicators in the period prior to diagnosis.
